# Delayed Failure after Endoscopic Staple Repair of an Anterior Spine Surgery Related Pharyngeal Diverticulum

**DOI:** 10.1155/2013/281547

**Published:** 2013-12-16

**Authors:** Samer Al-Khudari, Eric Succar, Robert Standring, Helmi Khadra, Tamer Ghanem, Glendon M. Gardner

**Affiliations:** ^1^Rush University Medical Center, Department of Otolaryngology Head and Neck Surgery, 1611 W. Harrison, Suite 550, Chicago, IL 60612, USA; ^2^Wayne State University School of Medicine, 42 West Warren Avenue, Detroit, MI 48202, USA; ^3^Henry Ford Health System, Department of Otolaryngology Head and Neck Surgery, 2799 W. Grand Boulevard, Detroit, MI 48202, USA; ^4^Tulane University School of Medicine, Department of Surgery, 1430 Tulane Avenue, New Orleans, LA 70112, USA

## Abstract

We present a rare complication of endoscopic staple repair of a pharyngeal diverticulum related to prior anterior cervical spine surgery. A 70-year-old male developed a symptomatic pharyngeal diverticulum 2 years after an anterior cervical fusion that was repaired via endoscopic stapler-assisted diverticulectomy. He initially had improvement of his symptoms after the stapler-assisted approach. Three years later, the patient presented with dysphagia and was found to have erosion of the cervical hardware into the pharyngeal lumen at the site of the prior repair. We present the first reported case of late hardware erosion into a pharyngeal diverticulum after endoscopic stapler repair.

## 1. Introduction

Diverticula that develop in the pharynx and esophagus are the result of abnormal traction or pulsion forces. Traction diverticula result from a pulling on the pharynx secondary to external force usually due to inflammation. Pulsion diverticula result from mechanisms that cause increased intraluminal pressure. Otolaryngologists most commonly encounter the pulsion type of diverticulum in Killian's triangle, which is located at the junction of the transverse fibers of the cricopharyngeus muscle (CP) and the oblique fibers of the lower inferior constrictor muscle. A diverticulum in this area was first described by Zenker and von Ziemssen in 1877 and is now commonly referred to as a Zenker's diverticulum [1]. The increased pharyngeal pressure is thought to result from a dysfunction in the relaxation of the upper esophageal sphincter or CP muscle. Presenting symptoms include dysphagia, halitosis, regurgitation of food, and weight loss. Currently, the most common treatments for a Zenker's diverticulum include conservative management, open surgical diverticulectomy (OSD) with CP myotomy, and stapler-assisted endoscopic diverticulectomy (SAED) with CP myotomy that was first reported by Collard et al. in 1993 [2]. The benefits of SAED over an open surgical technique include decreased hospital stay, decreased surgical time, and earlier initiation of oral feeding as reported by Smith et al. [3].

In 1991, Goffart et al. described the development of a pharyngeal diverticulum following anterior cervical spine surgery [4]. At the time of preparation of this paper, 12 cases of this finding had been reported in the literature [5]. These cases describe irregularly shaped diverticula, retropharyngeal fibrosis, and posterior esophageal adhesions suggesting that they are of the traction type. The presenting symptoms in these cases are commonly dysphagia, regurgitation, weight loss, fevers, cough, and choking typically occurring within three years of anterior cervical discectomy and fusion (ACDF). The most common intervention performed for these diverticula is open surgical diverticulectomy with CP myotomy. Two of the 12 reported cases underwent endoscopic stapler repair similar to the technique performed for a true Zenker's diverticulum. The present case reports a serious complication of endoscopic staple repair of a spine surgery related diverticulum in which the patient had hardware eroding into the pharyngeal lumen. We discuss the presentation, treatment options, and surgical management.

## 2. Case Report

**Figure 1:**
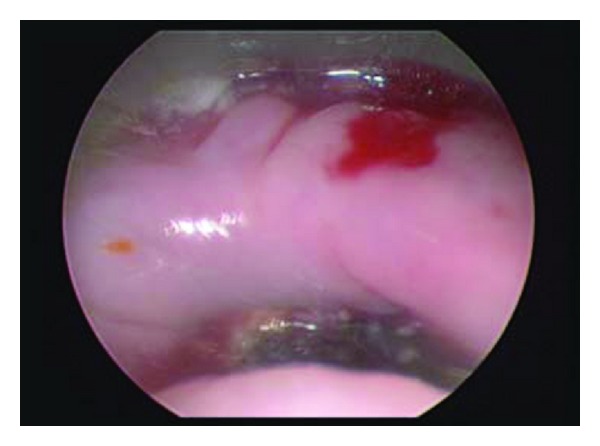
Intraoperative esophagoscopy demonstrating exposed cervical hardware and cricopharyngeal lip.

**Figure 2:**
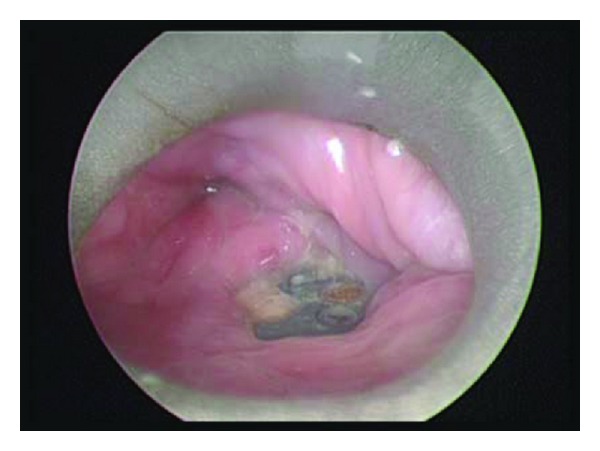
Intraoperative esophagoscopy demonstrating exposed cervical hardware.

**Figure 3:**
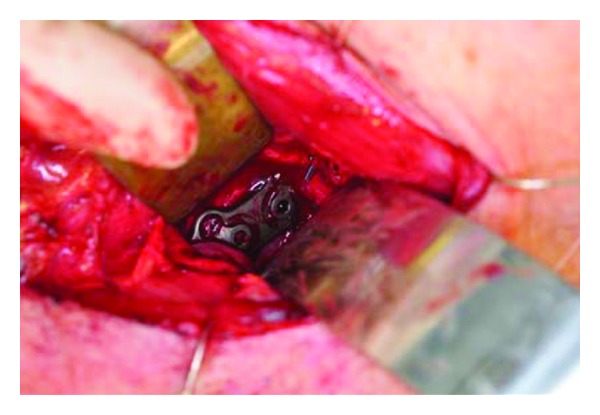
Intraoperative external cervical view of hardware.

**Figure 4:**
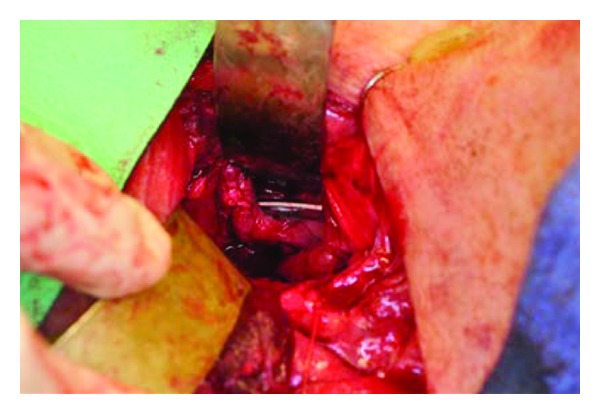
Intraoperative external cervical view of removed hardware and posterior esophageal tissue/scar band.

**Figure 5:**
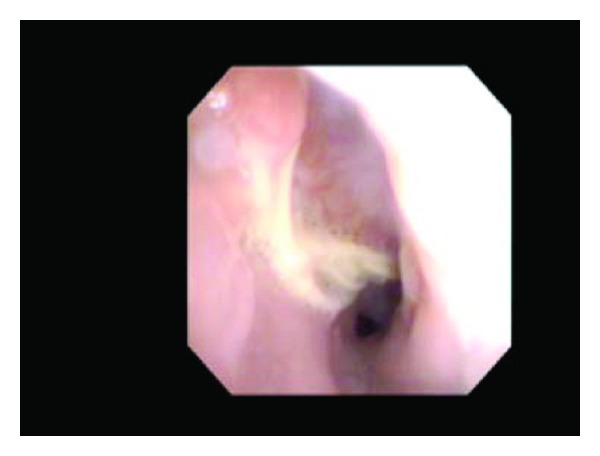
Six-week postoperative esophagoscopy picture of the healed radial forearm fascial flap and esophageal inlet.

**Figure 6:**
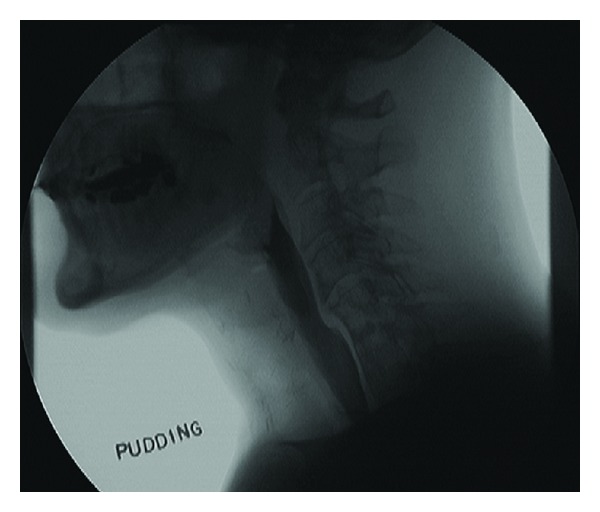
Esophagram 18 months after surgery depicting a patent lumen.

A 70-year-old male presented with symptoms of dysphagia two years after undergoing ACDF at the level of C5-C7. The esophagram at that time revealed a diverticulum. This was repaired one year later via an endoscopic stapler-assisted approach with complete resolution of symptoms. Thirty-five months later, the patient presented with dysphagia and endoscopy revealed exposure of the cervical hardware (Figure 1). Swallow imaging did not reveal active extravasation outside of the pharyngeal tract lumen (Figure 2). The patient subsequently underwent open cervical approach diverticulectomy with CP myotomy and hardware removal at C5-C7. A left forearm fascia free flap was placed to achieve a vascularized layer between the spine and esophageal closure and to promote healing (Figure 3). Intraoperative quantitative culture was negative for any organisms. Postoperatively, the patient had a mild infection treated with oral antibiotics and mild subcutaneous emphysema localized to the neck. The patient was maintained on tube feedings distal to the surgical site for 5 weeks via a gastrostomy. A dynamic swallow study performed six weeks postoperatively revealed excellent flow of barium through the esophagus with no evidence of a diverticulum or leak (Figure 4). The free flap and mucosal surfaces were well healed during that visit as evidenced by Figure 5. The patient was then returned to an oral diet without difficulty, and at 1 year postoperatively he continued to be without any significant complications. Repeat flexible endoscopy and esophagram at 18 months revealed an irregular but patent esophageal lumen (Figure 6).

## 3. Discussion

Dysphagia is a common complaint with many possible etiologies. Rarely, the cause is a pharyngoesophageal diverticulum [4]. The presence of an underlying esophageal motility disorder is the most common cause for a pharyngoesophageal diverticulum to develop, and it occurs more commonly in elderly males [6]. Pharyngeal diverticula that develop secondary to ACDF are often due to scarring from the hardware rubbing against the posterior aspect of the pharynx during swallowing. These diverticula commonly develop within 3 years after ACDF. In the past 2 decades, the prevalence of anterior cervical spine surgery for the treatment of degenerative cervical spine disease has increased by over 200% [7].

Dysphagia and hoarseness are well known complications of ACDF [3]. Early dysphagia may result from pharyngeal and pharyngoesophageal retraction intraoperatively, laryngeal intubation, recurrent laryngeal nerve injury, or infection [2]. Late onset dysphagia after ACDF is usually related to esophageal strictures, hardware movement, or malunion and occurs more commonly in patients undergoing revision cervical spine surgery. Dysphagia attributed to pharyngeal diverticula after ACDF is a rare occurrence; therefore there is no general consensus on treatment [2].

Ba, Allis, and Sood identified the physical findings of a pharyngeal diverticulum after ACDF to be dense scar tissue and adhesions in the prevertebral fascia around the diverticula [2, 5, 8]. This delayed esophageal injury following ACDF is often attributed to erosion from a screw migrating and piercing the pharynx. Yet, loose hardware is not necessary to cause esophageal injury. There have been reports of perforations due to compression and microtrauma associated with severe cervical osteophytes [4]. Delayed recognition of esophageal perforations can be dangerous as this may result in mediastinitis and possible death. In our case the cervical hardware did not have evidence of acute infection as the quantitative culture was found to have no growth, nor were there any signs of displaced hardware. Traction forces between the hardware and tissue causing scarring are the presumed reason for the pharyngeal diverticulum in our patient.

In the largest review of a diverticulum after ACDF, Allis et al. reported 3 new cases of diverticula and reviewed the prior 9 reported cases. In one of their newly reported cases, esophagoscopy showed that there was a mucosalized diverticulum with a thin cover overlying the spinal fusion hardware [5]. In our case, the cervical hardware eroded completely through the posterior wall of the diverticulum while maintaining an intraluminal seal at its lateral edges preventing leakage of saliva and contents into the neck and mediastinum.

There are various treatment options for patients with pharyngeal diverticula. Endoscopic stapling diverticulectomy has been reported as an effective procedure with low patient morbidity and a shorter hospital stay for Zenker's diverticulum; however, this is not necessarily the case for other pharyngeal diverticula. Several authors have noted inability to complete a successful staple repair due to the increased tissue thickness and scarring which is not usually present in a Zenker's diverticulum. Limited spine flexibility, poor pharyngeal exposure, and atypical shape of the diverticulum are other reasons why endoscopic staple repair may not be advised for a post-spine-surgery related diverticulum. If the underlying inciting agent is not removed (i.e., the hardware), then a similar inflammatory process may develop. In our case there was compromised tissue integrity after undergoing stapling of the vessels and tissue.

In the present case, we removed the existing hardware while performing an open approach diverticulectomy with CP myotomy and then reinforced the repair with a left forearm fascia free flap. This combination of procedures allowed for excellent exposure, removal of the hardware, and closure of the defect in the posterior wall of the diverticulum. The fascia flap provided a vascularized tuft of tissue that would prevent direct contact of the pharyngeal closure with the anterior surface of the cervical vertebrae and promote healing by bringing a robust blood supply to the affected area. This was also thought to assist in spontaneous closure of a fistula if one was to develop. Free tissue fascia and fascia-cutaneous flaps have been used with encouraging results for pharyngoesophageal reconstruction and are increasingly utilized in the salvage setting (i.e., with prior chemoradiation treatment) after total laryngectomy and partial laryngeal surgery [9]. Decreased fistula rates and improved fistula closure with conservative measures have been demonstrated in patients with prior chemotherapy and radiation undergoing pharyngeal reconstruction using free tissue transfer or pedicled flaps compared to primary closure [10].

In conclusion, as patients undergo increased spine surgery, it is important for otolaryngologists to consider the presence of traction in patients with a history of spine surgery who develop pharyngeal or esophageal diverticula. Until future larger studies are performed, the ideal treatment of a traction pharyngeal diverticulum is not known, but given the current research one must consider the potential for late failure after an endoscopic staple repair. Also, the surgeon must consider the need for removing the inciting hardware when possible.
